# The importance of the debate on occupational risk factors of COVID-19 for dental professionals

**DOI:** 10.31744/einstein_journal/2024CE1217

**Published:** 2024-10-21

**Authors:** Érika Mageste de Almeida Candido, Carlos Magno da Costa Maranduba, Melissa Mariana Gómez Vaca, Marcos Henrique de Castro e Souza, Warley Oliveira Silva, Antônio Márcio Resende do Carmo

**Affiliations:** 1 Universidade Federal de Juiz de Fora Faculdade de Odontologia Juiz de Fora MG Brazil Faculdade de Odontologia, Universidade Federal de Juiz de Fora, Juiz de Fora, MG, Brazil.; 2 Universidade Federal de Juiz de Fora Instituto de Ciências Biológicas Juiz de Fora MG Brazil Instituto de Ciências Biológicas, Universidade Federal de Juiz de Fora, Juiz de Fora, MG, Brazil.; 3 Universidad Nacional del Oriente Santa Cruz de la Sierra Santa Cruz Bolivia Universidad Nacional del Oriente, Santa Cruz de la Sierra, Santa Cruz, Bolivia.

Dear Editor,

The article titled "Factors underlying the high occupational risk of healthcare personnel for COVID-19 infection,"^(1)^ published in this journal, contributes to the discussion on the occupational risk of SARS-CoV-2 contamination among health professionals and the relevance of the application of infection-prevention and control measures in routine care.

Bodily fluids have a high potential for SARS-CoV-2 transmission. The detectable concentration of the virus in early stages of the disease in saliva,^(2)^ coupled with the ease of its collection, renders saliva of diagnostic value to detect SARS-CoV-2.^(3)^ If, on one hand, saliva has great diagnostic value, on the other hand, it has a high potential for transmission. Therefore, dental professionals should have the same, if not greater, level of concern as other health professionals, as in their routine clinical practice, they are exposed to highly contaminating fluids such as blood and, especially, saliva (without exception), by direct, indirect (contamined objects and surfaces) and aerosol contact.^(4)^

## References

[1] Crispim PM, Kawagoe JY, Rosseti AC, Menezes FG (2024). Factors underlying the high occupational risk of healthcare personnel for COVID-19 infection. einstein (São Paulo).

[2] Zhang W, Du RH, Li B, Zheng XS, Yang XL, Hu B (2020). Molecular and serological investigation of 2019-nCoV infected patients: implication of multiple shedding routes. Emerg Microbes Infect.

[3] Xu R, Cui B, Duan X, Zhang P, Zhou X, Yuan Q (2020). Saliva: potential diagnostic value and transmission of 2019-nCoV. Int J Oral Sci.

[4] van Doremalen N, Bushmaker T, Morris DH, Holbrook MG, Gamble A, Williamson BN (2020). Aerosol and Surface Stability of SARS-CoV-2 as Compared with SARS-CoV-1. N Engl J Med.

